# The case for home monitoring in hypertension

**DOI:** 10.1186/1741-7015-8-55

**Published:** 2010-09-27

**Authors:** Paul L Padfield

**Affiliations:** 1 Department of Medical Sciences, University of Edinburgh, Edinburgh, UK

## Abstract

Although the assessment of cardiovascular risk in individual patients takes into account a range of risk factors, the diagnosis and management of hypertension (high blood pressure) is largely determined by a single numerical value, albeit that often several readings are taken over time. Given the critical impact of a decision to embark on lifelong drug therapy, the importance of ensuring that a blood pressure (BP) record is both accurate and representative is clear. However, there is good evidence that the variability of BP is such that even if measurement is of the highest quality, it can be difficult to say with confidence whether a patient is above or below a treatment threshold. This commentary argues that current BP measurement is inadequate to make the clinical decisions that are necessary and that multiple readings are required to deliver an acceptable degree of accuracy for safe decision-making. This is impractical in a doctor's surgery, and the only realistic long-term strategy is to involve the patient in measuring his or her own BP in their own environment. Evidence is presented that such a strategy is better able to predict risk, is cost-effective for diagnosing hypertension, can improve BP control and is thus better able to protect individuals in the future.

In this commentary, I explain why doctors and other healthcare professionals should increase their familiarity with the technology, be aware of its strengths and limitations and work with patients as they become more empowered in the management of their chronic condition, hypertension.

## Introduction

Hypertension is the most important risk factor for cardiovascular disease in the world [1], and evidence that lowering blood pressure reduces cardiovascular risk has been substantiated by arguably the greatest body of randomised control trial data in clinical medicine.

Given the vast amount of information relating to hypertension in the world literature, it is interesting to reflect on relatively how little importance has been paid to the measurement of blood pressure itself. A cursory glance at many of the landmark trials over the past 25 years will show that there is no consistency in the documentation of the measurement of blood pressure, nor how many readings are taken to establish BP levels at a given time. Particularly surprisingly, some studies do not even detail how blood pressure was measured.

The measurement of blood pressure with a mercury sphygmomanometer is now more than 100 years old and although many primary care doctors in the United Kingdom have moved to the use of aneroid devices, it is the mercury manometer that has been the mainstay of clinical trials. There is no doubt that if properly maintained and used correctly, this device will record an accurate measurement of blood pressure at any point in time.

One would hope and believe that in clinical trials, great care is taken to measure blood pressure accurately, but outside the confines of a trial we know that terminal digit preference whereby readings end in either '0' or '5' are commonplace and the impact of the relationship between the measurer and the measured can be considerable, giving rise to what has come to be called 'white coat hypertension' in its most extreme form (see below).

One of the difficulties imposed on us in clinical practice is that whilst we use data from clinical trials to inform our decisions, doctors are always considering the management of individual patients who may well behave quite differently from those in trials.

## Variability of Blood Pressure

**Figure 1:**
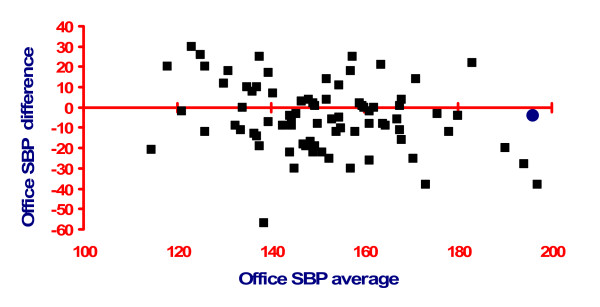
**Variability of systolic blood pressure (BP) on repeated measurement (research study conditions) taken from **[16]**with permission (Padfield PL: Self-monitored blood pressure: a role in clinical practice? ***Blood Press Monit *2002, 7(1):41-44.) A Bland Altman plot relating the average of two separate BP readings, taken 2 weeks apart (*x*-axis), against the difference between those two readings (*y*-axis) in 85 subjects where there were no treatment changes between readings. Note the mean similarity between the two occasions (blue circle) but the enormous individual variability.

Many doctors may be unaware of the enormous variability of blood pressure in an individual subject. This is such that differences in blood pressure between successive visits to a surgery or clinic might simply relate to random variation rather than the impact of treatment. Even in the carefully controlled environment of a clinical trial, where trained nurses measure blood pressure, differences across 2 weeks can be as much as 30 mmHg with no treatment changes (see Figure 1).

Despite the fact that we appreciate that blood pressure conveys risk in a continuous fashion, we have developed cut points to define hypertension in different clinical scenarios. The most common accepted definition of hypertension for most individuals is 140/90 mmHg. If a patient's blood pressure is measured at 250/180 mmHg, then it is likely that however many times this blood pressure is measured, it will always be high but if the figure is much closer to the point of definition (140/90), it is not difficult to see that the diagnosis of hypertension may not be robust.

Some years ago, our group analysed data from the placebo arm of the Medical Research Council's first treatment trial of mild hypertension (involving many thousands of patients) and demonstrated this lack of consistency [2]. If one used a diastolic BP (DBP) of 90 mmHg to define hypertension after 3 months of observation in that trial, then that diagnosis would be maintained in 80% of subjects after a further 3 months of observation. Conversely, if after 3 months DBP was < 90 mmHg (i.e., normal), 30% could be hypertensive 3 months later. In routine clinical practice, patients labelled as hypertensive after several measurements are likely to be started on treatment and any drop in blood pressure thereafter attributed to the effects of the drug. While such observations may have little impact on the outcome of a clinical trial, they will have considerable impact on how we manage individual patients.

It has recently been stated that the variability of blood pressure itself may be important in determining outcome [3], but conventional management still depends on the assumption that the blood pressure measured gives a fair representation of an individual's 'usual' BP. It should be clear from the above that this view is difficult to sustain, and to be certain of an individual's true usual BP one would need to obtain multiple measurements to increase confidence in categorising someone's BP as normal or not [4].

It is difficult to believe that this is practicable, given the way health care is currently delivered, and we need to consider alternatives if we require many measurements of BP.

## Out-of-Office Blood Pressure

The development of microelectronics first necessitated by the NASA space programme has resulted in new technologies for measuring blood pressure. Assessment of the waveform produced by blood flowing through arteries can be made oscillometrically, and devices now exist which can convert such waveforms into a single measurement of blood pressure equivalent to that obtained using a stethoscope listening to Korotkov sounds. This has led to two similar but different ways of obtaining multiple blood pressure measurements.

### Ambulatory Blood Pressure Monitoring

While beyond the scope of this commentary, the use of devices that can be worn by patients over (usually) a 24-hour period with multiple measurements has demonstrated a number of points:

1. The average of multiple measurements at home tends to be lower that that measured in a surgery or clinic, although this difference is less if the blood pressure is measured by a nurse rather than by a doctor [5].

2. The reproducibility of the average produced is much greater than that of clinic measurements (see ref. [2]).

3. Observational data have demonstrated that such measurements are much better predictors of cardiovascular outcome than any clinic or surgery BP. Arguably, this is simply because the figure produced by the average of multiple measurements is a more robust measure of an individual's usual pressure.

Ambulatory monitors remain expensive, probably are best utilised in centres of expertise and, whilst cost-effective for the diagnosis of hypertension, are not practicable for the long-term management of individuals who require changes in drug therapy over months and years.

### Self- or Home Blood Pressure Monitoring

There remains variability in the literature as to what to call this technology, and although the term 'home monitoring' is still used extensively, such devices can be used in any environment.

The science that led to the development of ambulatory monitors is available in small, simple-to-use self-monitors that use the same oscillometric technology and allow patients to measure blood pressure themselves semiautomatically as often as they wish or as required.

Home monitors are generally at least an order of magnitude cheaper than ambulatory devices and can be bought without reference to a health care professional in major pharmacies in the United Kingdom and many other countries.

As such electronic devices have developed; national specialist organisations have recommended that they are validated against the so-called 'gold standard' of the mercury manometer and a variety of validation protocols have been published to ensure that retailed machines are indeed accurate.

It is salutary to note that there is no obligation on a manufacturer to demonstrate accuracy before selling their devices, and there are examples of inaccurate machines being sold to the public. In Great Britain and Ireland, there are important agencies that have realised this problem and maintain a web-based list of validated devices for both patients and healthcare professionals [6,7].

The average blood pressure obtained by multiple measurements with a self-monitor approximates to the daytime average of an ambulatory monitor [8], and there are some who have argued that self-monitoring can displace an ambulatory monitor in all aspects of hypertension diagnosis and management. What most home monitors will still not do is measure blood pressure through the night, and it is worth mentioning in passing that there is evidence that nocturnal pressure has independent predictability in terms of cardiovascular outcomes. As we do not yet have any clinical strategies as to how to treat nocturnal pressure, this remains an area for further research rather than a practical clinical reality.

## Who uses self-monitors?

This is different in different parts of the world, and there are data to suggest that some 85% of patients with hypertension in Japan will have purchased a machine. The figure is around 65% in the USA [9] but probably as low as 10-30% in the United Kingdom [10]. Most machines are bought by patients without instruction or guidance from a healthcare professional, and we have little information as to how they are used to either help or hinder the management of individual patients, particularly within the United Kingdom.

Anecdotally, it is clear that patients will look at the wide range of blood pressures and learn very quickly that blood pressure does not stay the same. If they are not properly educated, they can assume that the machines are inaccurate, and certainly they receive no consistent response when they attend the general practitioner, who may record a much higher blood pressure in the clinic.

## Management with self-monitored readings

Most international hypertension guidelines suggest the use of clinic measurements to guide management and make little mention of the variability of blood pressure outlined above. Where they mention the use of ambulatory or self-blood pressure monitoring, they are careful to emphasise the importance of using such information in conjunction with that obtained by clinic measurements, although how this is to be done is often not defined.

As long as we hold to a definition of 140/90 as the threshold for defining hypertension, it is worth emphasising that for both an average daytime ambulatory pressure and the average of a set of home-monitored readings, this equates roughly to around 135/85.

**Figure 2:**
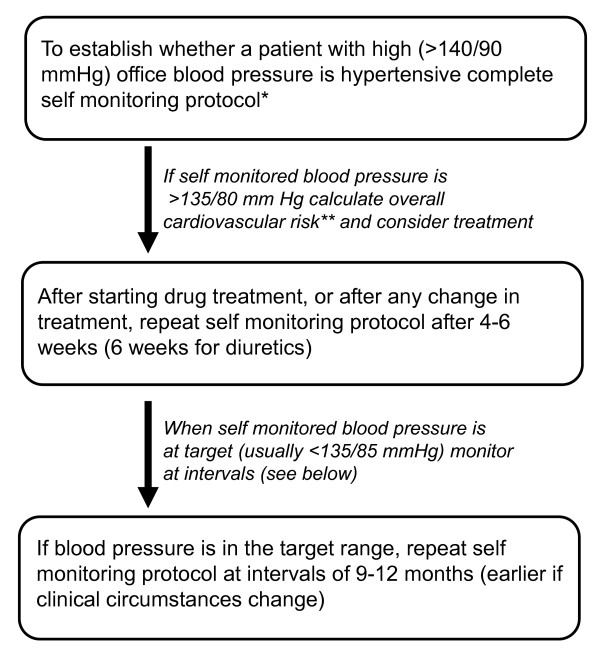
**Suggested model for self-monitoring of blood pressure, adapted from **[11]**(BMJ Publishing Group Ltd. Copyright 2008) Whilst there is no clear evidence base to prefer this model, it will deliver an average of 24 readings and has been adopted by the European Society of Hypertension **[17]**, the American Society of Hypertension and the American College of Cardiology (see **[9]**)**. *The protocol should consist of a series of measurements over 7 days with two measurements morning and evening. The first day's data should be discarded and the remaining 24 measurements averaged. Any management decision must be preceded by monitoring, such that at least 10 measurements are available for averaging. **Cardiovascular risk is calculated using equations that incorporate office blood pressure measurements, not self-monitored readings.

There are a number of algorithms and guidelines available as to how one might use self-monitoring of blood pressure to diagnose and manage hypertension, and they all depend upon obtaining a set of readings before making a clinical decision. Once one averages 20-25 readings, the figure derived is at least twice as reproducible as that in the clinic and is practicable for most patients [11] (see Figure 2).

## Cautions

Not every patient will benefit from the use of self-monitors. Whilst newer monitors can detect abnormal rhythms and indicate that there is such a clinical situation, their accuracy is questionable in this context. It should be noted, however, that it is difficult to measure blood pressure accurately by any methodology in someone with atrial fibrillation, particularly if there is a fast ventricular response, but it is probably not suitable to use electronic, oscillometric devices in the presence of significant cardiac arrhythmias.

It is often cited that patients may be disturbed by measuring their own blood pressure and may become introverted and obsessed by the readings. We have little idea as to what proportion of the patient population might suffer in this way, but any guideline to patients would need to take this into account and consider the impact it would have on them as individuals.

There is a natural temptation for patients to measure their blood pressure at times of stress or during any unexpected event, and it is important to emphasise to them that blood pressure will rise under such circumstances but that that should not be considered a problem and should not normally require a change in therapy. It is the average of many measurements rather than the single odd reading that should govern the introduction or a change in drug therapy.

## Evidence for benefit

Many of the early trials testing whether the use of self-monitoring improved blood pressure control in patients were too small to truly answer the question, and while some were positive, others showed no evidence of benefit. A meta-analysis by Cappuccio some years ago showed that there was evidence of a small but significant improvement in blood pressure control for those patients who were given a self-monitor [12].

If giving a self-monitor is accompanied by input from a healthcare professional such that treatment is affected by the readings obtained more directly, then the evidence of benefit is much more clear-cut [13,14], and there are several trials underway examining the potential for longer-term blood pressure control using this simple methodology.

Within our own group, we are researching the value of telemetry such that patients are given a self-monitor with blood pressure readings instantly transmitted wirelessly to nurses in a healthcare centre who, on the basis of a 'rolling average', will guide treatment along predetermined algorithms of care. A recently published trial has shown positive results [15].

The term 'patient empowerment' is used a lot in modern healthcare literature, and governments are also keen on patients taking responsibility themselves for some of the management of chronic conditions. Hypertension is a classic case in point because it is not an illness, simply a risk factor, and generally speaking treatment changes are based on the figures obtained rather than on any symptoms. If patients can understand their blood pressure more, see their own measurements, and see the impact of treatment, then arguably they may be more likely to comply with medical therapy in the longer term, even if the treatment does not appear to be making them feel better.

The evidence for this is scarce currently, and again trials are underway to assess whether this is a reality.

Whether one is a convert to the use of self-blood pressure monitoring or not, it is difficult to argue against the fact that such machines are being used extensively by patients themselves in primary care. We can choose to ignore the results that they obtain or we can work with them to improve the information about blood pressure levels and thus perhaps produce more effective drug treatment for the condition of hypertension that is the cause of so much morbidity and mortality.

## Competing interests

The authors declare that they have no competing interests.

## Pre-publication history

The pre-publication history for this paper can be accessed here:

http://www.biomedcentral.com/1741-7015/8/55/prepub
